# Comparison Between Autologous Albumin Gel and Liquid Platelet-Rich Fibrin Mixture Versus Connective Tissue Graft to Modify the Gingival Phenotype: A Randomized Controlled Trial

**DOI:** 10.7759/cureus.61958

**Published:** 2024-06-08

**Authors:** Sara Abdulhak, Tarek Kassem, Yasser Alsayed Tolibah

**Affiliations:** 1 Periodontology, Damascus University, Damascus, SYR; 2 Pediatric Dentistry, Damascus University, Damascus, SYR

**Keywords:** keratinized tissue width, gingival thickness, gingival phenotype, connective tissue graft, albumin gel-platelet-rich fibrin mixture

## Abstract

Objectives

To assess changes in gingival thickness (GTH) and the width of keratinized gingival tissue (KTW) following treatment with either connective tissue graft (CTG) or an albumin gel-platelet-rich fibrin mixture (Alb-PRF).

Materials and methods

Twenty treatment sites were included in a split-mouth design involving 10 patients with a thin gingival phenotype in the mandibular anterior region. The sample was randomly divided into two groups, with the Alb-PRF applied to the experimental group and CTG used for the control group. GTH and KTW were measured at baseline and after one, three, and six months.

Results

GTH increased in both groups during all follow-up periods. However, no statistically significant differences (p > 0.05) between the groups were observed at baseline and six months. At three months, the experimental group exhibited significantly higher GTH (p < 0.001). Additionally, at three and six months, the CTG group showed a superior increase in KTW (p < 0.05).

Conclusion

Within the constraints of this study, Alb-PRF application for modifying thin gingival phenotypes proved to be an effective therapeutic option, potentially serving as an alternative to CTGs. Although Alb-PRF resulted in thicker gingiva, CTG demonstrated a greater enhancement in KTW.

## Introduction

The term "periodontal phenotype" encompasses the phenotypic traits of the soft tissues and bone that constitute the periodontium [1]. The World Workshop on the Classification of Periodontal and Peri-Implant Diseases and Conditions, held in 2017, introduced a new approach for periodontal conditions comprising two components: the bone morphotype (bone morphology) and the gingival phenotype (three-dimensional gingival volume). For the bone morphotype, measurements of buccal bone plate thickness (BBPT) are taken, while keratinized tissue width (KTW) and gingival thickness (GTH) are measured for the gingival phenotype [2].

For over 30 years, platelet concentrations (PCs) have been utilized in dentistry as a regenerative tool capable of releasing supra-physiological doses of growth factors [3]. There is strong evidence that PCs can act as an autologous source of growth factors and healing cytokine biomolecules, including platelet-rich plasma (PRP), platelet-poor plasma (PPP), and platelet-rich fibrin (PRF) release, to promote and ensure the healing of both soft and hard tissues in the oral region.

However, previous generations of these concentrates faced challenges. Firstly, before processing PRP, anticoagulants need to be added to prevent blood coagulation, which has been shown to inhibit healing [4]. Another challenge was the short lifespan of PRP, with the majority of growth factors being metabolized within hours to days. The short in vivo turnover rate of PRF has been one of its key drawbacks [5].

To overcome these challenges, the albumin gel-platelet-rich fibrin mixture (Alb-PRF) was developed, which increased the longevity of a traditional PRF membrane's capacity for regeneration [6]. Recent studies have shown that by heating a liquid PPP layer, heated albumin's (albumin gel's) resorption characteristics can be prolonged from two weeks to more than four months [5].

This mechanism creates a flexible barrier that traps cells and platelets and is made of dense protein complexes wrapped in fibrin fibers [7]. It has proven to be highly stable structurally for 21 days in mouse subcutaneous tissue [8], and it may release growth factors and cytokines gradually [8,9]. Furthermore, due to interactions with its proteins, albumin can carry many drugs, making it a promising candidate for drug delivery roles because of these three particular domains [10,11].

There is a wealth of research supporting the use of albumin in tissue engineering [12]. This is due to its great purity, homogeneity, quantity, and simplicity in isolating from blood plasma precipitation [13].

Albumin-enriched biomaterials exhibit low decline over time, indicating reduced in vitro degradation, and offer an ideal structure for cell growth [12]. Additional research indicates that the fibrin network's ultrastructure may be stabilized by the connection with albumin [13]. Furthermore, early results show that adding denatured serum albumin greatly improves the PRF-based scaffold, producing an autologous, biocompatible substance that may have improved durability and prolonged activity [5,8,11].

Currently, there are relatively few therapeutic options available in the literature to prevent further clinical/surgical consequences of thin phenotypic, such as gingival recession. The development of a preventative approach to thickening gingiva could prevent invasive surgical operations and the anticipated disadvantages associated with a thin phenotype. The use of Alb-PRF in thin gingival tissue is a minimally invasive, affordable, painless, and side-effect-free technique. Therefore, this study aims to compare Alb-PRF membrane and connective tissue graft (CTG) (the gold standard for treating areas that need gingival augmentation and root coverage) [14] to modify the thin gingival phenotype. The null hypothesis suggests that there is no significant difference in GTH and KTW after modifying the thin gingival phenotype using CTG or Alb-PRF.

## Materials and methods

Study design, settings, and ethical approval

This randomized, split-mouth, single-blinded clinical trial aimed to compare two treatment approaches (CTG and Alb-PRF) in modifying the gingival phenotype, employing a superiority design with a 1:1 allocation ratio. The study was conducted at Damascus University's Faculty of Dentistry, Department of Periodontology, from September 2021 to June 2022, ensuring adherence to the ethical principles outlined in the Declaration of Helsinki through the study design, questionnaires, and informed consent process. Approval for the research project was obtained from the Local Research Ethics Committee of the Faculty of Dentistry, Damascus University (UDDS-02082021/SRC-2659). The study was funded by Damascus University (Funding No.: 501100020595) and was registered at the International Standard Randomised Controlled Trial Number (ISRCTN) registry under the ID ISRCTN18042063 on 24/04/2023. The revised CONSORT (Consolidated Standards of Reporting Trials) statement guided the development of this randomized clinical trial.

Recruitment and eligibility criteria

During the study period, 50 patients aged between 18 and 30 years were referred to the Department of Periodontology due to thin gingival morphologies in their lower anterior teeth. The principal researcher (SA) conducted an examination to identify healthy individuals with mandibular anterior teeth gingival thickness less than 1 mm. The gingival thickness was measured by a #25 endodontic spreader (Mani, Inc., Tochigi, Japan) and a caliper (Mitutoyo, Kanagawa, Japan). A preoperative clinical assessment was performed to measure gingival thickness and the width of keratinized tissue. Thirty-four patients met this criterion. Twenty-four individuals were excluded due to systemic disorders compromising their overall immunological status, smoking habits, contraindications to orthodontic intervention, malocclusion, crowding, or issues involving missing or supernumerary teeth. All patients who agreed to participate signed an informed consent form after being fully briefed on the trial and its treatment component.

Sample size calculation

Based on the data of a previous study [15], the sample size in the present study was calculated by G*Power 3.1.9.4 (Heinrich-Heine-Universität, Düsseldorf, Germany). In the ANOVA study, sample sizes of 20 surgical sites were obtained from the two groups within 10 patients as a split-mouth design (each patient had two surgical sites, one for the Alb-PRF group and one for the CTG group). This sample size achieved 80% power to detect differences with a 0.05 significance level. To account for potential withdrawals, the sample size was increased to 12 patients (24 surgery sites).

Randomization

The mandibular anterior region's two sides were randomly designated for one of two procedures: A (left: Alb-PRF, right: CTG) or B (right: Alb-PRF, left: CTG). The allocation sequence was executed using a computer random generator with a 1:1 allocation ratio. Opaque-sealed envelopes, identified by the patient's initials, concealed the allocation sequence. Envelopes corresponding to each patient were opened before surgery. Y.A.T., who did not contribute to patient treatment and measuring the outcomes, made the randomization and generated the sequence.

Blinding

The study was conducted as a single-blinded trial, where the treating periodontist could not be blinded to the employed technique during treatment due to the nature of the interventional trial. In the subsequent data analysis, the outcome assessors (two pre-trained Ph.D. students at the Department of Periodontology, Damascus University) remained uninformed about the patient's allocation.

Clinical procedures

Pre-surgical Phase

Following patient selection, each participant received comprehensive oral hygiene instructions. Root debridement and scaling procedures were conducted. Surgical intervention for altering the gingival phenotype was deferred until the participant demonstrated a high level of plaque control. Plaque and gingival indices for each patient were meticulously documented on a custom examination card [16].

Surgical Phase

Recipient bed preparation periosteum: The recipient bed was prepared using a tunneling method, beginning with the application of local anesthesia (Huons Lidocaine HCL, Seoul, Korea) to the recipient area. A single incision, made with a No. 15 blade (Swann Morton, Sheffield, UK), extended from the midpoint of the gingival papilla base distally to the sides of the lower canine, reaching the mucogingival junction on both the right and left sides. Careful elevation of the full-thickness flap was performed using a periosteal elevator (Artman, Acworth, Georgia), ensuring no incisions were made in the gingival papillae or vestibular areas, until detachment on each side reached 1 mm before the midline between the lower incisors. In the experimental group, autologous albumin gel mixed with liquid platelet fibrin-rich was used, while the control group received a connective graft.

Preparation of autologous Alb-PRF: Whole blood samples were collected in sterile conical-bottom plastic tubes (15 mL; Qingdao Carong Import & Export Co., Ltd., Qingdao, China) without any additives. Two tubes were then placed in a centrifuge (33° rotor angulation and 86 mm at the maximum; Laboratory Centrifuge EBA 200 series, Andreas Hettich GmbH & Co. KG, Tuttlingen, Germany) and centrifuged at 2700 rpm and 75°C for 12 minutes (~708 × g) to create Alb-PRF membrane.

Using a syringe fitted with an 18 G needle, approximately 2 ml of the first section of plasma was extracted, while the remaining blood, containing a high concentration of red blood cells, was left at room temperature (20°C).

The syringes containing PPP were then placed inside a Bio-Heat (Bio-PRF, Venice, FL) apparatus, designed to denature proteins using human plasma. After 10 minutes at 75°C (as per the manufacturer's instructions), the syringes were allowed to cool at room temperature for an additional 10 minutes. Subsequently, 4 ml of the rich fraction from the buffy coat layer was collected and added to the heated PPP layer in a glass container. Gentle mixing was performed using a 10 ml syringe with an 18 G needle. Once the fibrin polymerization process was complete, which typically took about five minutes, the membrane was formed.

Harvesting the connective tissue graft and securing it: The connective graft was harvested from the palate's dome using the full-thickness technique. Bleeding points, equivalent in size to the required connective graft, were strategically positioned in the molar and premolar regions, away from the marginal gingiva on the palatal side, to prevent recession or other complications. Incisions measuring 1 to 1.5 mm deep were then made to connect these points, allowing for the extraction of a graft containing both epithelium and connective tissue together (partial thickness). Subsequently, deepithelialization was performed using a sharp 15 or 15C blade parallel to the outer surface of the graft. Healing occurred through secondary intention, and the donor site wound was covered by securing gelfoam (Surgispon, Aegis Lifesciences, Ahmedabad, India) with an X-stitch (Shanghai Pudong Jinhuan, Shanghai, China). Once the sutures were removed, the connective graft was fully secured with absorbable sutures in the tunnel area, ensuring no part of it protruded outside the flap. Finally, the released incision was closed with non-absorbable sutures.

Postsurgical Instructions

During the initial phases of the surgical procedure and as necessary thereafter, prescriptions for 500 mg of amoxicillin (capsules) and 600 mg of Brufen were provided. Patients were instructed to gently cleanse the treatment area with moist cotton soaked in a 0.12% chlorhexidine solution instead of brushing their teeth in that region for two weeks. Subsequently, atraumatic tooth brushing techniques were employed to maintain plaque control. Sutures were removed after seven days.

Outcomes measures

The patients were recalled for clinical examination at the end of the first week, after a month, three months, and six months.

Gingival Thickness Index

Gingival thickness was measured using a no. 15 endodontic spreader (inserted perpendicularly from the vestibular midpoint 1.5 mm apical of the gingival margin through the soft tissue until a hard surface is reached) and a digital caliper to assess the penetration depth.

Width of Keratinized Tissue Index

Keratinized tissue width was measured from the gingival margin to the mucogingival junction using a periodontal probe (UNC 15 probe; Medesy, Maniago, Italy).

**Figure 1:**
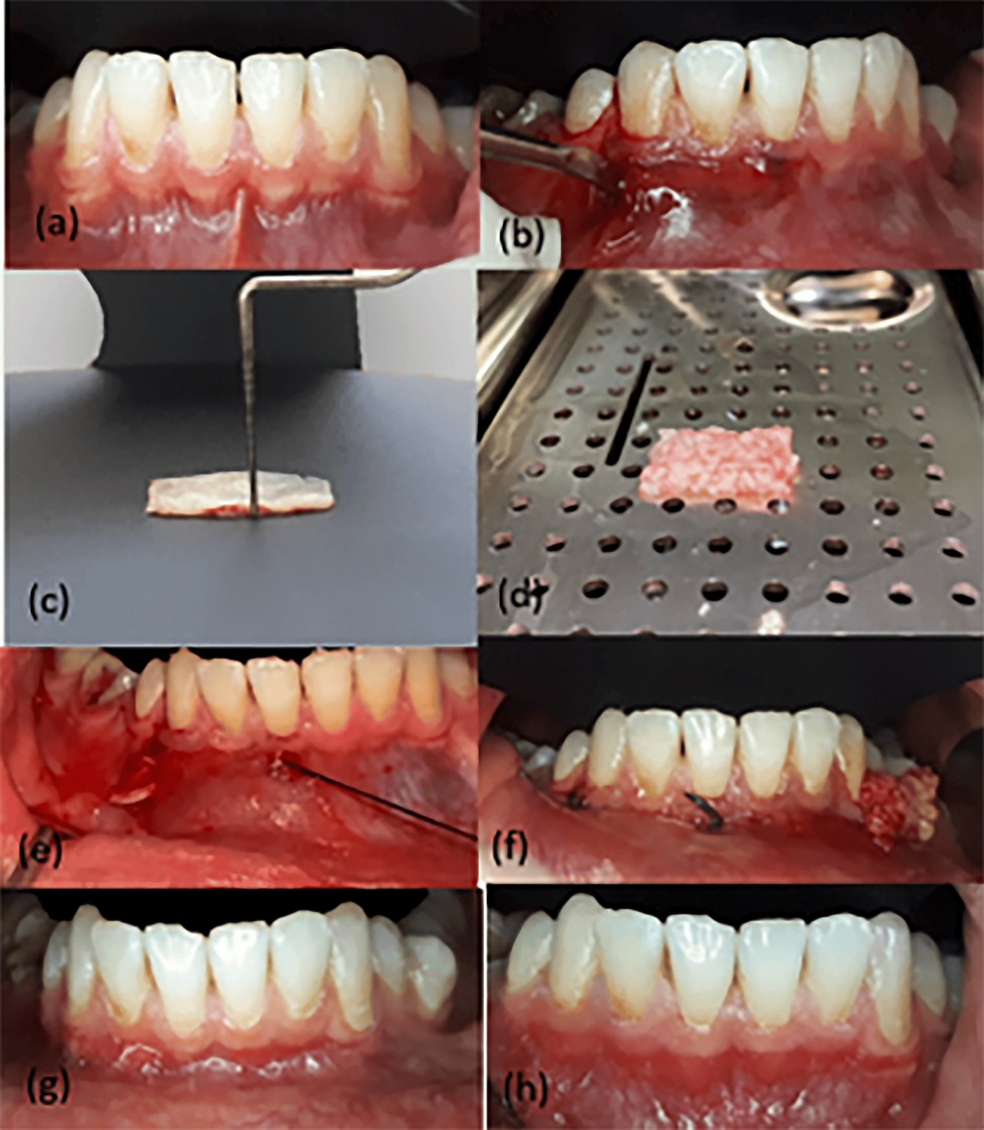
A case of thin gingival phenotype (the left side received Alb-PRF treatment, while the right side received CTG treatment). (a) The case before the surgery. (b) Full-thickness flap detaching. (c) The connective tissue graft. (d) Alb-PRF membrane. (e) Suture of connective tissue graft. (f) The Alb-PRF being interred into the tunnel. (g) The results after three months follow-up. (h) The results after six months follow-up. Alb-PRF: albumin gel-platelet-rich fibrin mixture; CTG: connective tissue graft.

Figure 1 summarizes the procedure steps and the healing observed in the follow-up periods.

Two blinded pre-trained Ph.D. students at the Department of Periodontology, Damascus University did the outcomes measurement procedure.

Statistical analysis

Utilizing SPSS (version 17.0 for Windows, SPSS Inc., Chicago, IL), data analysis was done by a blinded statistician who was unaware of the group procedure. The Shapiro-Wilk test was used to determine whether the data distribution was normal. The distribution of all the parameters was found to be normal. At each assessment time, the two groups were compared using the unpaired t-test to find significant differences.

## Results

**Figure 2:**
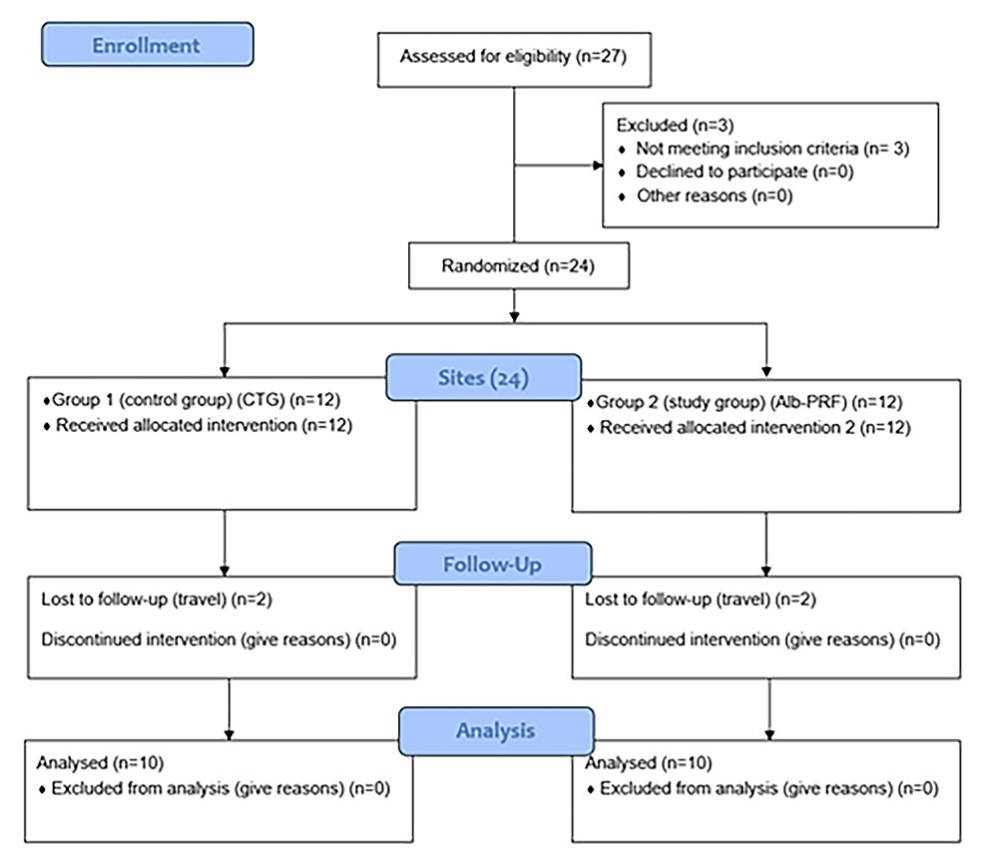
Flow chart of the study. Alb-PRF: albumin gel-platelet-rich fibrin mixture; CTG: connective tissue graft.

Two patients were lost to follow-up; therefore, 10 participants with a thin gingival phenotype (six females and four males) were included in the investigation. The patients' average age was 28 years. Figure 2 shows the patient flowchart for this study.

In each group, clinical parameters were assessed at baseline, at three months, and at six months follow-up. The unpaired t-test was used to compare the mean KTW at baseline, three months, and six months between the Alb-PRF and CTG groups (control).

In comparison to the Alb-PRF group, the mean KTW at three and six months was considerably higher in the CTG group. The mean KTW at baseline did not differ significantly (Table 1). The unpaired t-test was used to compare the mean GTH between the CTG (control) and Alb-PRF groups at baseline, three months, and six months. While there was no discernible change in the mean GTH at baseline or after six months between the Alb-PRF and CTG (control) groups, there was a significant difference in the mean GTH at three months between the Alb-PRF and CTG (control) groups, and it was significantly higher in the Alb-PRF group (Table 2). After six months, there were no appreciable differences in GTH between the two groups (p > 0.05). After six months, the control group's KTW was superior to that of the Alb-PRF group (3.25 ± 0.67 and 2.8 ± 0.71, respectively; p = 0.003).

**Table 1:** Keratinized tissue width comparison between the groups.

Keratinized tissue width	Connective tissue graft (control)		Albumin gel-platelet-rich fibrin mixture		Mean difference	T-test value	P-value
	Mean	Standard deviation	Mean	Standard deviation			
Baseline	2.6 mm	0.65828 mm	2.75 mm	0.79057 mm	-0.15 mm	-1.964	0.081
3 months	3.25 mm	0.66667 mm	2.8 mm	0.71492 mm	0.45 mm	4.07	0.003
6 months	3.25 mm	0.66667 mm	2.8 mm	0.71492 mm	0.45 mm	4.07	0.003

**Table 2:** Gingival thickness comparison between the groups.

Gingival thickness	Connective tissue graft (control)		Albumin gel-platelet-rich fibrin mixture		Mean difference	T-test value	P-value
	Mean	Standard deviation	Mean	Standard deviation			
Baseline	0.717 mm	0.14937 mm	0.719 mm	0.13820 mm	-0.002 mm	-0.254	0.805
3 months	1.7 mm	0.14614 mm	2.0820 mm	0.22195 mm	-0.382 mm	-14.329	0.000
6 months	1.699 mm	0.14723 mm	1.693 mm	0.15456 mm	0.006 mm	0.325	0.752

## Discussion

Gingival phenotype has garnered significant attention in periodontal histology in recent years, owing to its substantial impact on the outcomes of various dental treatments such as orthodontic, prosthetic, regenerative, and dental implant procedures [17]. Different clinical factors and interactions can lead to diverse behaviors in gingival phenotypes. The thick phenotype is often preferred over the thin phenotype due to its greater resilience to trauma, inflammation, and subsequent recession, making it more manageable and predictable in dental procedures [18,19]. Several surgical techniques, including autologous gingival grafts, acellular dermal matrix, PRF membranes, and fetal membrane, have been explored in the literature to modify the gingival phenotype [20,21] Moreover, minimally invasive methods such as micro-needling (MN) were included in recent studies [22,23], where several sessions of injectable PRF injections combined with MN in thin gingiva were reported to augment GTH. While CTG is considered the gold standard for gingival augmentation and root coverage, it is associated with significant pain, particularly in palatal graft harvesting [24].

Platelet concentrates offer a consistent stimulus for wound healing, but traditional PRF treatments have limitations such as low volume and rapid resorption. Integrating autologous platelet gels with other biomaterials holds promise for enhancing regeneration [25]. In this study, Alb-PRF was proposed as a self-acceptable alternative to connective grafts, aiming to mitigate the discomfort and pain associated with graft harvesting. Comparing Alb-PRF to CTG using the tunnel technique for modifying thin gingival phenotypes represents a novel approach in this clinical trial.

Alb-PRF, the latest iteration of PRF, addresses the shortcomings of previous versions and exhibits qualities ideal for facial aesthetics, regeneration, or repair. Its albumin component undergoes processing that densifies its structure and prolongs resorption while maintaining higher concentrations of platelets, leukocytes, and growth factors compared to other PRF types [26]. Employing a split-mouth design and transgingival probing for quantitative assessment ensured the reliability and repeatability of outcomes [27,28]. The tunnel approach, by avoiding vertical release incisions, maintains sufficient blood flow at the recipient site, resulting in superior outcomes [29].

GTH and KTW were the primary outcomes of interest in this study. While both groups showed an increase in GTH during follow-up periods, significant differences were observed at three months [29]. Moreover, the difference in KTW between the CTG and Alb-PRF groups was statistically significant at three and six months [30]. SCTG demonstrated exceptional biomimetic ability in increasing the keratinized gingival strip, highlighting its potential in inducing keratinization of the gingival mucosa and enhancing periodontal connective tissue adhesion [15].

Despite being the first study to evaluate Alb-PRF for modifying gingival phenotype in a clinical model, limitations such as a lack of comparable studies and a relatively small sample size underscore the need for further research. Another limitation is the short follow-up period. Nonetheless, within the scope of this clinical trial, Alb-PRF showed promising results for modifying gingival phenotype, warranting exploration across various surgical applications.

Further histological and long-term clinical research with larger sample sizes is required to enhance the understanding of the mechanism of Alb-PRF in improving the thin gingival phenotype.

## Conclusions

Despite the limitations outlined in the study, it is evident that both surgical techniques resulted in improved GTH six months post surgery. However, the control group demonstrated superior KTW compared to the Alb-PRF group. These findings suggest that Alb-PRF is a promising therapeutic option for addressing the thin gingival phenotype and may serve as a viable alternative to CTG.
